# AI-driven CT assessment of erector spinae myosteatosis: muscle quality and its association with strength and frailty in older surgical patients

**DOI:** 10.3389/fmed.2026.1935672

**Published:** 2026-10-07

**Authors:** Moritz F. Mayr, Thierno D. Diallo, Christoph Maurer, Moritz M. M. Albrecht, Tobias Scheef, Alexander Rau, Marco Reisert, Fabian Bamberg, Hagen Schmal

**Affiliations:** 1 Department of Orthopedics and Trauma Surgery, University Medical Center Freiburg, Freiburg, Germany; 2 Department of Diagnostic and Interventional Radiology, Faculty of Medicine, Medical Center - University of Freiburg, University of Freiburg, Freiburg, Germany; 3 Department of Neurology and Neuroscience, Faculty of Medicine, Medical Center, University of Freiburg, Freiburg, Germany; 4 Division of Medical Physics, Department of Diagnostic and Interventional Radiology, Faculty of Medicine, University Medical Center Freiburg, University of Freiburg, Freiburg, Germany; 5 Department of Orthopedic Surgery and Traumatology, Odense University Hospital, Odense, Denmark

**Keywords:** body composition, deep learning, frailty, muscle quality, myosteatosis, sarcopenia

## Abstract

**Background:**

Frailty and sarcopenia are overlapping syndromes that increase adverse outcomes in older adults. Computed tomography (CT) is a reference standard for body composition analysis, but manual segmentation is time-consuming. This exploratory study used a fully automated deep learning model (DLM) to investigate associations between CT-derived muscle quality and quantity and clinical frailty.

**Methods:**

We performed a retrospective, cross-sectional secondary analysis of 53 geriatric surgical patients (mean age 77.4 ± 4.4 years). A DLM segmented skeletal muscle (SM), subcutaneous and visceral adipose tissue at the third lumbar vertebral level. Intermuscular adipose tissue (IMAT) was quantified exclusively within the erector spinae muscle and is therefore reported as a surrogate marker of trunk muscle quality rather than whole-muscle myosteatosis. Frailty was assessed with the Clinical Frailty Scale (CFS), handgrip strength and the Timed Up and Go (TUG) test. Because functional testing was incomplete, effective sample sizes varied (*n* = 21–53) and are reported for every model.

**Results:**

In the total cohort, a higher IMAT Index was associated with lower handgrip strength (*ρ* = −0.434, *p* = 0.004, *n* = 43) and remained independent after adjustment for age, sex, BMI and ASA status (B = −1.808, 95% CI −2.994 to −0.622, *p* = 0.004, *n* = 42). Its association with the CFS was borderline univariately (*ρ* = 0.273, *p* = 0.051, *n* = 52) and absent after adjustment (*p* = 0.184, *n* = 51). Neither index was independently associated with TUG performance (*p* = 0.134 and *p* = 0.071, *n* = 25). In an exploratory sex-stratified analysis, the IMAT Index correlated with the CFS in women (*ρ* = 0.727, *p* < 0.001, *n* = 19) but not in men. Because the female subgroup was small (*n* = 20), no sex-stratified regression or interaction test was performed.

**Conclusions:**

In this small, retrospective, cross-sectional cohort, an erector-spinae-based IMAT Index was associated with handgrip strength but not independently with global clinical frailty. Automated opportunistic CT analysis may therefore identify specific muscle weakness rather than global frailty. The observed sex differences are hypothesis-generating and require confirmation in larger, adequately powered longitudinal studies.

## 1 Introduction

Frailty is defined as a clinically recognizable state of increased vulnerability that extends beyond the normal physiological ageing trajectory, resulting from accelerated declines in reserve and function across multiple physiologic systems (1, 2). This condition predisposes individuals to a higher risk of adverse health outcomes, including falls, hospitalization, and mortality. Frailty constitutes a frequent pathology, affecting approximately 11% of community-dwelling older adults, with prevalence increasing significantly with age to approximately 51% among those aged 90 years or older (3, 4). Consequently, frailty has emerged as a key public health issue, substantially influencing healthcare resource utilization (5–7) Frailty is generally conceptualized through two main models: the phenotype model, focusing on physical signs such as exhaustion, weakness, and slowness, and the deficit accumulation model, which quantifies frailty as a cumulative index of health deficits (1, 8). Various screening instruments operationalize these conceptual models into clinical practice. Performance-based measures such as the Timed Up and Go (TUG) test primarily assess the physical phenotype through mobility evaluation, while handgrip strength quantifies muscle weakness—a cardinal feature of physical frailty (1, 2). In contrast, the Clinical Frailty Scale (CFS) integrates multiple domains to provide a global clinical judgment more aligned with the deficit accumulation approach (8, 9). Importantly, frailty is a dynamic process. Early identification is crucial as the condition is potentially reversible through targeted interventions (3, 10). Sarcopenia and frailty are distinct but overlapping syndromes (9, 11). While sarcopenia focuses specifically on muscle-related decline, it represents a key biological substrate underlying physical frailty (12). Studies suggest that up to 50% of frail older adults meet criteria for sarcopenia, highlighting their interconnection (11). Computed tomography (CT) at the level of the third lumbar vertebra (L3) is an established parameter for quantifying body composition (13, 14). However, muscle mass alone has frequently shown only a weak association with functional deficits. In contrast, muscle quality, specifically the presence of myosteatosis—characterized by intermuscular adipose tissue (IMAT)—has been reported to show a closer association with muscle function than muscle mass alone (15, 16). High levels of IMAT were consistently linked to reduced muscle strength and poor physical performance, independent of total muscle mass (17, 18). This occurs because intramuscular fat infiltration impairs muscle contractile function by disrupting muscle architecture (19, 20), alters metabolic signaling, and promotes local inflammation through the secretion of pro-inflammatory cytokines such as Interleukin-6 (IL-6) and TNF-alpha (21). Despite the clinical relevance of body composition parameters, manual segmentation of CT scans is time-consuming and prone to observer variability (15). Recent technological advances rely on automated Deep Learning Models (DLM) to precisely segment skeletal muscle (SM) and adipose tissue compartments (22, 23). This study investigated the correlation between these automated CT-derived parameters (IMAT, SM, SAT, VAT) and established frailty assessments (CFS, TUG and handgrip strength) in a geriatric cohort. We hypothesize that: (1) IMAT will show more pronounced correlations with frailty measures than muscle area alone, reflecting the importance of muscle quality over quantity in the assessment of age-related functional decline (23, 24) and (2) CT-derived metrics will demonstrate more pronounced associations with tests directly assessing muscle strength compared to those involving complex coordination or global functional judgment (TUG, CFS), as imaging parameters represent the morphological substrate of the muscle (12).

## 2 Material and methods

### 2.1 Study design and participants

We report a secondary retrospective analysis of the PAWEL study, a multicenter trial evaluating interventions to prevent postoperative delirium (25). Participants for this investigation were prospectively recruited between 2017 and 2019 at the University Medical Center Freiburg. Inclusion criteria for the parent study required participants to be ≥70 years of age and scheduled for elective surgery with an expected duration of at least 60 min. For this secondary analysis, we included individuals with an abdominal CT scan encompassing the L3 level performed within 3 to 12 months perioperatively. Exclusion criteria were defined as: (1) imaging data not sufficient for automated body composition assessment, (2) missing clinical assessment data, or (3) an inability to perform physical testing due to acute illness. This study was approved by the Ethics Committee of the University of Freiburg (Vote No. 474/17) and carried out in accordance with the Declaration of Helsinki and its later amendments. We followed the Strengthening the Reporting of Observational Studies in Epidemiology (STROBE) guidelines for observational studies.

### 2.2 Clinical frailty assessments

Clinical assessments were performed at baseline prior to surgery using established geriatric screening tools as part of the PAWEL study protocol:

- **CFS:** The overall frailty status was graded using the CFS. This 9-point scale relies on clinical judgment to integrate comorbidities, mobility, and functional independence, ranging from 1 (very fit) to 9 (terminally ill) (8). The CFS was assessed by trained geriatricians during the preoperative evaluation.
- **Handgrip Strength:** Isometric handgrip strength was measured using a calibrated handheld dynamometer. The procedure followed the standardized protocol described by Fried et al. (1). Measurements were taken with the patient seated and the elbow flexed at 90 degrees. The maximum value of three trials with the dominant hand was recorded in kilograms.
- **TUG test:** Functional mobility was assessed using the TUG test (26). The TUG test was performed in a standardized hospital corridor using a standard chair and a 3-meter walking distance marked on the floor. Patients were allowed to use their customary walking aid if needed. The best time of two trials was recorded in seconds.

### 2.3 CT image acquisition and body composition analysis

#### 2.3.1 Image acquisition, slice selection and deep learning-based segmentation

All patients underwent abdominal CT imaging as part of routine assessment for planned elective surgery. Examinations were performed using one of three multidetector scanners: SOMATOM Definition Flash, SOMATOM Definition Edge, or SOMATOM Force (Siemens Healthineers, Erlangen, Germany), with scan parameters consistent with standard clinical abdominal protocols. For body composition analysis, a single axial slice at the L3 level was selected, as this anatomical landmark is widely established for estimating whole-body SM and adipose tissue mass. The L3 level was identified at the mid-level of the vertebral body where both transverse processes were clearly visible and the vertebral body was complete without intervertebral disc space, ensuring high reproducibility (14, 15). To ensure consistent and time-efficient analysis, a fully automated DLM was utilized for tissue segmentation on this axial slice. The model architecture follows a hierarchical, multi-scale U-Net-type design as previously described (27). The development and validation of the DLM were performed as follows:

1. **Ground Truth Generation:** A training dataset consisting of 100 randomly selected, routinely acquired abdominal CT scans (varying contrast phases) was manually annotated by an experienced radiologist. Manual segmentation was performed using the web-based medical imaging platform NORA (https://www.nora-imaging.org). All segmentations were independently reviewed and adjusted by a board-certified radiologist.
2. **Tissue Definitions:** The manual annotation and subsequent automated segmentation differentiated between three main compartments based on expert visual assessment and characteristic anatomical features:
  a **Skeletal Muscle (SM):** This compartment included the psoas major, erector spinae, quadratus lumborum, and the abdominal wall muscles (transversus abdominis, obliquus internus and externus, rectus abdominis) (15, 27).
  b **Visceral Adipose Tissue (VAT):** Defined as adipose tissue enclosed by the inner SM wall and the anterior aspect of the vertebral column.
  c **Subcutaneous Adipose Tissue (SAT):** Defined as adipose tissue located between the skin and the outer SM fascia.
3. **Model Training:** The DLM was trained for pixel-wise classification to generate segmentation masks for SM, VAT, and SAT, optimizing the loss between predicted masks and expert annotations. Model outputs were compartment areas reported in dm^2^ (converted to cm^2^ for reporting).
4. **Independent Testing and Quality Control:** Performance was evaluated on an independent test set of 40 abdominal CT examinations that did not include any participant of the present study cohort. The DLM demonstrated high concordance with expert manual segmentation, achieving Dice coefficients of 0.97 ± 0.01 for SAT, 0.94 ± 0.06 for VAT and 0.97 ± 0.02 for SM, with Pearson correlation coefficients of r = 1.00 (*p* < 0.001) between manual and automated segmentations for all three compartments (27). These performance metrics refer to the segmentation of the main tissue compartments (SM, VAT, SAT). A separate quantitative validation of IMAT delineation within the erector spinae muscle is not available. The validation cohort consisted of routinely acquired abdominal CT examinations of adult patients and was not restricted to a geriatric surgical population, which should be considered when generalising the reported accuracy to the present cohort. All automated segmentations were visually inspected by a radiologist to ensure accuracy and identify potential segmentation errors.

#### 2.3.2 Quantification of myosteatosis

A key focus of this study was the precise quantification of myosteatosis via IMAT. In our automated analysis, IMAT was specifically defined and quantified as the adipose tissue located within the fascial boundaries of the erector spinae muscle, serving as a surrogate marker of trunk muscle quality rather than as a measure of whole-muscle myosteatosis (17). The DLM segmented this compartment to enable the calculation of cross-sectional areas for each specific tissue type. The process of automated segmentation and targeted IMAT quantification is illustrated in Figure 1. By comparing the original axial CT slice at the L3 level (Panel A) with the resulting DL segmentation mask (Panel B), the model's high precision in delineating intermuscular fat (pink) within the erector spinae muscle from the surrounding skeletal muscle compartments (blue) is demonstrated. This anatomical separation allows muscle quality to be assessed even when global muscle mass appears preserved. Because IMAT was quantified exclusively within the erector spinae, all IMAT results reported below refer to this specific compartment and should not be interpreted as whole-muscle myosteatosis.

**Figure 1:**
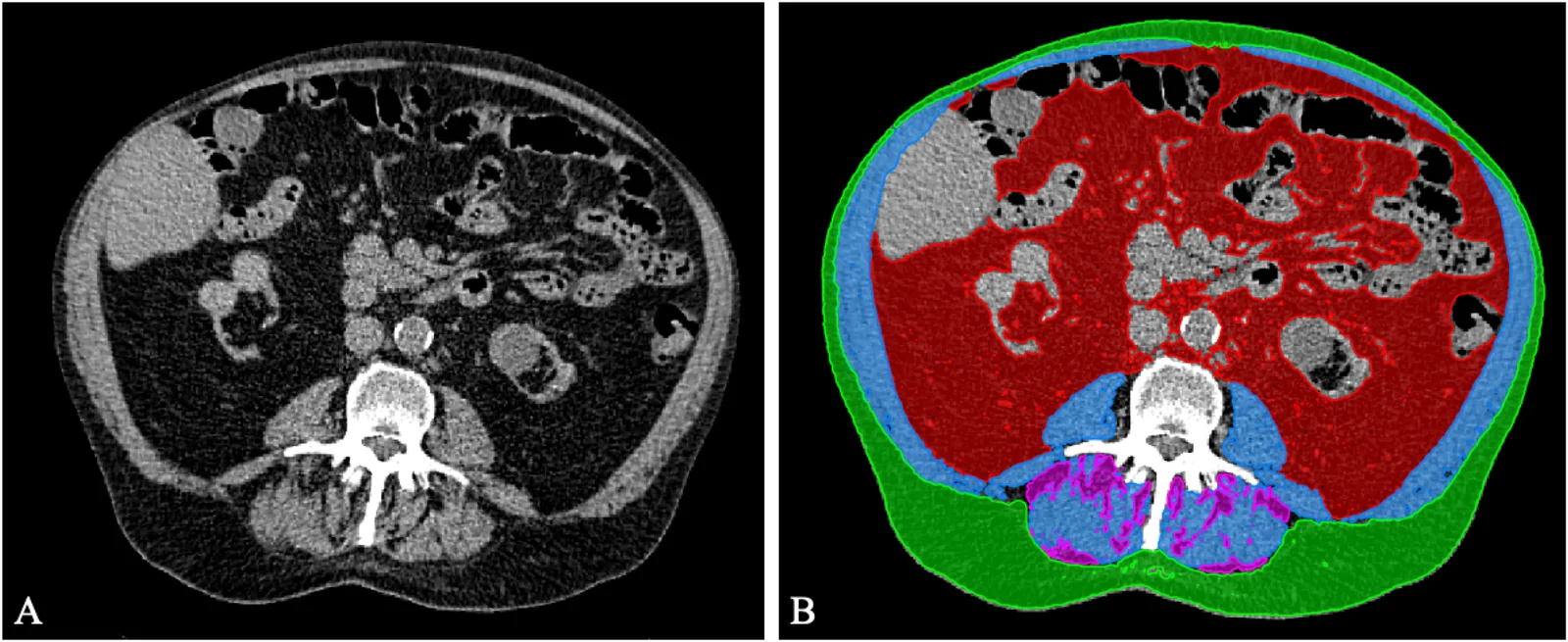
Deep-learning–based quantification of myosteatosis at the L3 vertebral level. **(A)** Representative original axial CT image at the L3 level utilized as input for the automated analysis. **(B)** Corresponding segmentation mask generated by the multi-scale U-Net DLM. The visualization highlights the distinct tissue compartments: SM (blue), IMAT within the erector spinae muscle (pink), VAT (red), and SAT (green).

### 2.4 Calculation of indices

To account for inter-individual differences in body stature, the absolute areas in cm² were normalized for patient height. The following indices were calculated and used for statistical analysis:

- **SM Index:** Total skeletal muscle area at L3/height^2^ (cm^2^/m^2^).
- **IMAT Index:** Total IMAT area at L3/height^2^ (cm^2^/m^2^).
- **SAT Index and VAT Index:** Respective adipose areas/height^2^ (cm^2^/m^2^).

These normalized indices serve as the primary variables for correlating body composition with clinical frailty parameters (28).

### 2.5 Statistical analysis

Statistical analysis was performed using IBM SPSS Statistics (Version 30.0; IBM Corp., Armonk, NY, USA). The Shapiro–Wilk test was used to assess the normality of data distribution. Descriptive data for normally distributed variables [including age, BMI (Body Mass Index), handgrip strength, and TUG] are expressed as mean ± standard deviation (SD). Variables showing a non-normal distribution (specifically the CT-derived parameters: IMAT Index, SM Index, SAT Index, and VAT Index) are presented as median with interquartile range (IQR). Categorical and ordinal variables [such as sex, CFS and ASA (American Society of Anesthesiologists) score] are presented as frequencies and percentages. To evaluate clinical differences across the frailty spectrum, baseline characteristics and functional performance measures are reported for the total cohort and compared between two subgroups: non-frail (CFS 1–4) and frail (CFS 5–9). Inter-group comparisons were performed using the Mann–Whitney U test for continuous variables and the Chi-squared test for categorical data. As part of quality control, all automated segmentation results were screened for physiological plausibility. In one patient the automated muscle segmentation failed, returning a skeletal muscle area of 0.03 cm^2^ (SM Index 0.01 cm^2^/m^2^) compared with a minimum of 74.7 cm^2^ in all other patients. This value was judged physiologically impossible and was set to missing; the patient was retained in the cohort for all analyses not involving the SM Index, because the corresponding clinical and adipose tissue measurements were valid. To investigate the relationship between body composition parameters and clinical frailty measures, Spearman's rank correlation coefficients (*ρ*) were calculated. Correlation coefficients were interpreted according to the thresholds proposed by Mukaka and by Schober et al., with coefficients below 0.40 in absolute value regarded as weak (29, 30). These analyses were performed for the total cohort and additionally stratified by sex to explore potential biological differences in body composition associations. The non-parametric approach was selected because the CT-derived parameters showed a significant deviation from normal distribution (*p* < 0.05 in Shapiro–Wilk test). Multiple linear regression analyses were subsequently conducted to determine the independent association of CT-derived metrics (independent variables) with clinical outcomes (dependent variables). Unlike the correlation analysis, regression models were calculated for the total cohort only. Due to the limited sample size of female participants (*n* = 20), a stratified regression analysis would have lacked sufficient statistical power to accommodate the required number of predictors, and no formal test for interaction between sex and body composition was performed. To adjust for essential clinical confounders despite the modest sample size, candidate predictors were evaluated in separate multivariable linear regression equations alongside a predefined block of covariates comprising age, sex, BMI, and ASA physical status. Each model therefore contained five predictors. For the handgrip and CFS models this corresponds to approximately eight to ten observations per predictor, whereas the TUG models contain only five observations per predictor. The latter fall below conventional recommendations and carry a substantial risk of overfitting and unstable coefficient estimates. For this reason, unstandardised regression coefficients are reported with 95% confidence intervals, adjusted R^2^ values are given alongside R^2^, and the effective sample size is reported for every correlation and regression model. Multi-collinearity was assessed using the Variance Inflation Factor; all values were below 2.2. Patients with missing data for any variable were excluded from analyses involving that variable (pairwise for correlations, listwise within each regression model). All statistical tests were two-tailed, and a *p*-value of < 0.05 was considered statistically significant. Given the exploratory nature of this analysis and the number of associations examined, no adjustment for multiple comparisons was applied and all *p*-values should be interpreted as descriptive.

## 3 Results

### 3.1 Patient characteristics and functional status

From the original multicenter trial cohort, a total of 53 geriatric patients met the eligibility criteria and were included in the final analysis (Figure 2).

**Figure 2:**
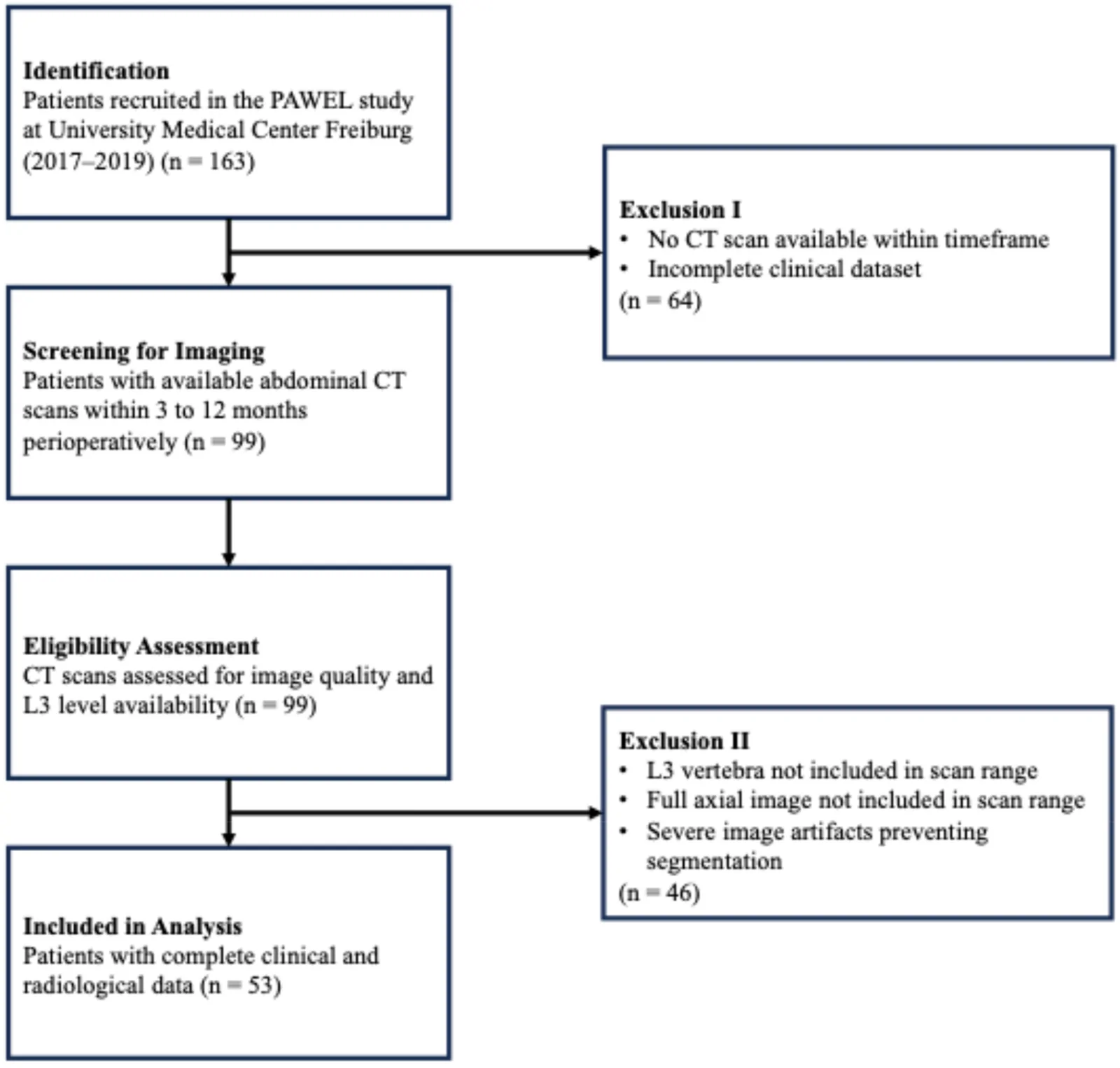
Study flow chart of the participant selection process.

The flowchart illustrates the recruitment and selection pathway for geriatric patients from the original PAWEL study population. From the 163 patients initially recruited at the University Medical Center Freiburg, 64 patients were excluded due to the lack of available imaging or incomplete clinical datasets (Exclusion I). 99 were screened for this secondary imaging analysis based on the availability of a perioperative abdominal CT scan within a 3 to 12-month timeframe. Following a detailed eligibility assessment of those an additional 46 patients were excluded because the L3 vertebra or the full axial image was not entirely within the scan range, or severe image artifacts prevented reliable automated segmentation (Exclusion II). The final study cohort included in the statistical analysis comprised 53 patients with complete clinical and radiological data.

The study cohort comprised 53 patients with a mean age of 77.4 years and a predominantly male distribution (Table 1). Clinical frailty, as measured by the CFS, showed a median score of 4.0, reflecting a vulnerable study population (3, 8). Based on the established cut-off, the participants were nearly equally distributed between the non-frail (CFS 1–4; *n* = 28) and frail (CFS 5–9; *n* = 25) groups. In the assessment of functional performance, significant differences were observed across the frailty spectrum. Patients in the frail group demonstrated significantly slower mobility in the TUG test compared to their robust counterparts (*p* = 0.035), highlighting the objective physical decline associated with higher CFS scores. Furthermore, a clear clinical trend toward reduced muscle strength was observed in the frail group, as evidenced by lower handgrip strength values, although this difference did not reach formal statistical significance (*p* = 0.073). Demographic parameters, including age and BMI, were comparable between the two groups, suggesting that the observed functional differences were primarily driven by frailty status rather than age or body mass alone.

**Table 1:** Baseline demographics, clinical characteristics, and physical performance measures for the total cohort and subgroups stratified by frailty status.

Variable	Total (*N* = 53)	Robust (CFS 1–4, *n* = 28)	Frail (CFS 5–9, *n* = 25)	*p*-value*
Age (years), mean ± SD	77.4 ± 4.4	76.6 ± 4.0	78.4 ± 4.7	0.105
Sex (Female), *n* (%)	20 (37.7%)	11 (39.3%)	9 (36.0%)	> 0.99
BMI (kg/m^2^), mean ± SD	25.4 ± 4.3	25.1 ± 4.4	25.8 ± 4.3	0.471
CFS score, median (IQR)	4.0 (3.2–4.8)	3.2 (3.0–3.5)	5.0 (4.5–6.0)	**< 0.001**
TUG (s), mean ± SD	13.2 ± 4.7 (*n* = 26)	12.1 ± 4.6 (*n* = 18)	15.8 ± 4.2 (*n* = 8)	**0**.**035**
Handgrip strength (kg), mean ± SD	26.0 ± 7.8 (*n* = 44)	28.4 ± 6.2 (*n* = 24)	23.2 ± 8.7 (*n* = 20)	0.073

Data are presented as mean ± SD for normally distributed variables or median (IQR) for non-normally distributed data. The effective sample size is given where functional testing was incomplete. *p-values represent inter-group comparisons between robust and frail participants using the Mann–Whitney U test for continuous variables and the Chi-squared test for categorical data. Statistically significant *p*-values (< 0.05) are highlighted in bold.

### 3.2 CT-derived body composition

The automated analysis of CT imaging data provided detailed indices for muscle mass and various adipose tissue compartments. In one patient the automated muscle segmentation returned a physiologically implausible result and was therefore treated as missing, so that SM Index values were available for 52 of 53 patients. When comparing the robust and frail subgroups, no statistically significant differences were observed in any of the CT-derived body composition parameters. The SM Index and the indices for SAT and VAT remained comparable across both groups, indicating a similar baseline distribution of muscle quantity and regional adiposity.

Although the IMAT Index was higher in frail participants compared to their robust counterparts, this difference did not reach statistical significance in the current cohort (*p* = 0.229). These findings suggest that while there are clinical trends toward higher intermuscular fat infiltration in frail individuals, the compartmental indices alone do not provide a clear distinction between the frailty groups in this sample. Detailed values for all imaging metrics are presented in Table 2.

**Table 2:** CT-derived body composition parameters of the study cohort stratified by frailty status.

Parameter	Total (*N* = 53)	Robust (CFS 1–4, *n* = 28)	Frail (CFS 5–9, *n* = 25)	*p*-value*
SM Index (cm^2^/m^2^), median (IQR)	46.75 (41.19–52.13) *n* = 52	47.01 (43.45–50.54) *n* = 28	46.12 (39.36–52.25) *n* = 24	0.776
IMAT Index (cm^2^/m^2^), median (IQR)	2.11 (1.23–3.35) *n* = 52	1.91 (1.09–2.83) *n* = 28	2.55 (1.57–4.11) *n* = 24	0.229
SAT Index (cm^2^/m^2^), median (IQR)	46.36 (32.41–67.70) *n* = 53	47.12 (35.19–71.64) *n* = 28	43.06 (29.63–67.49) *n* = 25	0.612
VAT Index (cm^2^/m^2^), median (IQR)	51.19 (25.34–77.24) *n* = 52	41.16 (20.08–86.17) *n* = 28	53.15 (37.72–65.52) *n* = 24	0.963

Data are presented as median (IQR) with the effective sample size for each cell. SM Index values were available for 52 of 53 patients because one automated muscle segmentation returned a physiologically implausible result and was set to missing; IMAT and VAT Index values were available for 52 patients owing to missing segmentation data in one patient. *p-values represent inter-group comparisons between robust and frail participants using the Mann–Whitney U test.

### 3.3 Correlations between body composition and clinical frailty

Spearman rank correlation analysis revealed distinct associations between body compartments and physical performance (Table 3).

**Table 3:** Spearman’s rank correlation matrix between CT-derived body composition parameters and clinical frailty assessments.

Variable	IMAT Index	SM Index	SAT Index	VAT Index	TUG	Handgrip Strength	CFS
IMAT Index	1.000						
SM Index	*ρ* = −0.125 *p* = 0.379 *n* = 52	1.000					
SAT Index	*ρ* = 0.497 ***p*** **<** **0.001** *n* = 52	*ρ* = −0.087 *p* = 0.542 *n* = 52	1.000				
VAT Index	*ρ* = 0.383 ***p*** **=** **0.005** *n* = 52	*ρ* = 0.217 *p* = 0.123 *n* = 52	*ρ* = 0.505 ***p*** **<** **0.001** *n* = 52	1.000			
TUG	*ρ* = 0.115 *p* = 0.585 *n* = 25	*ρ* = 0.133 *p* = 0.526 *n* = 25	*ρ* = −0.237 *p* = 0.245 *n* = 26	*ρ* = 0.083 *p* = 0.694 *n* = 25	1.000		
Handgrip Strength	*ρ* = −0.434 ***p*** **=** **0.004** *n* = 43	*ρ* = 0.302 ***p*** **=** **0.049** *n* = 43	*ρ* = −0.321 ***p*** **=** **0.034** *n* = 44	*ρ* = 0.006 *p* = 0.972 *n* = 43	*ρ* = −0.279 *p* = 0.221 *n* = 21	1.000	
CFS	*ρ* = 0.273 *p* = 0.051 *n* = 52	*ρ* = 0.003 *p* = 0.984 *n* = 52	*ρ* = 0.006 *p* = 0.968 *n* = 53	*ρ* = 0.141 *p* = 0.317 *n* = 52	*ρ* = 0.492 ***p*** **=** **0.011** *n* = 26	*ρ* = −0.253 *p* = 0.098 *n* = 44	1.000

Data are presented as Spearman's rank correlation coefficients (ρ) with the corresponding *p*-value and the effective sample size (n) for each pairwise comparison. Sample sizes differ between cells because functional assessments were incomplete in part of the cohort and because one implausible SM Index value was set to missing. CT-derived metrics include height-normalised indices (cm^2^/m^2^) for IMAT, SM, SAT and VAT. Clinical assessments comprise functional mobility (TUG), isometric muscle strength (handgrip strength) and the CFS. Coefficients are interpreted according to Mukaka and Schober et al., with absolute values below 0.40 regarded as weak. *P*-values < 0.05 are considered statistically significant and are highlighted in bold.

#### 3.3.1 Muscle quality and quantity

An inverse correlation was found between the IMAT Index (as a marker of lower muscle quality/myosteatosis) and muscle strength. A higher IMAT Index was significantly associated with lower isometric handgrip strength (*ρ* = −0.434, *p* = 0.004, *n* = 43), indicating that lower muscle quality accompanies lower strength. In comparison, the quantitative SM Index showed a positive correlation with handgrip strength that only marginally reached statistical significance (*ρ* = 0.302, *p* = 0.049, *n* = 43). Following the interpretation thresholds proposed by Mukaka and by Schober et al., a coefficient of this magnitude is regarded as weak, whereas the IMAT correlation is of moderate magnitude (29, 30). The difference between the two coefficients was not formally tested.

#### 3.3.2 Adipose tissue compartments (SAT & VAT)

Regarding the global adipose tissue depots, the SAT Index showed a negative correlation with handgrip strength (*ρ* = −0.321, *p* = 0.034, *n* = 44), mirroring the direction of the IMAT association, although a coefficient of this magnitude is likewise regarded as weak (29, 30). In contrast, the VAT Index showed no significant correlation with handgrip strength or the TUG test in the total cohort.

#### 3.3.3 Functional mobility and frailty score

Results differed by assessment tool. None of the CT-derived parameters (indices of IMAT, SM, SAT and VAT) correlated significantly with TUG performance, for which functional data were available in only 25 to 26 patients. A positive correlation of weak magnitude was observed between the IMAT Index and the CFS, which did not reach statistical significance (*ρ* = 0.273, *p* = 0.051, *n* = 52).

#### 3.3.4 Sex-stratified analysis

In an exploratory analysis stratified by sex, correlations between body composition and frailty measures were observed in women but not in men. In the female subgroup (*n* = 20), the IMAT Index correlated with the CFS score (*ρ* = 0.727, *p* < 0.001, *n* = 19) and with handgrip strength (*ρ* = −0.594, *p* = 0.015, *n* = 16). The VAT Index also correlated positively with the CFS score in women (*ρ* = 0.516, *p* = 0.024, *n* = 19). None of these associations reached statistical significance in the male subgroup. Given the small size of the female subgroup, the absence of a sex-stratified regression analysis and the fact that no formal test for interaction between sex and body composition was performed, these results are exploratory and do not demonstrate a sex-specific effect.

### 3.4 Predictors of frailty: multivariable regression analysis

To determine the independent association of muscle quality versus muscle quantity with clinical frailty parameters, separate multiple linear regression models were calculated. All models were adjusted for age, sex, BMI and ASA physical status. Unstandardised coefficients are reported with 95% confidence intervals, and the effective sample size is given for each model (Table 4). Given the modest number of observations per model, and particularly for the TUG models (*n* = 25, corresponding to five observations per predictor), these estimates are imprecise and should be regarded as exploratory.

**Table 4:** Multivariable linear regression models predicting clinical frailty assessments.

Outcome variable	Predictor of interest	*n*	B (95% CI)	β	*p*-value	R^2^ (adjusted R^2^)
Handgrip strength	IMAT Index	42	−1.808 (−2.994 to −0.622)	−0.443	**0**.**004**	0.517 (0.449)
	SM Index	42	0.062 (−0.268 to 0.393)	0.065	0.703	0.391 (0.306)
TUG	IMAT Index	25	0.993 (−0.336 to 2.322)	0.328	0.134	0.336 (0.161)
	SM Index	25	−0.293 (−0.614 to 0.028)	−0.505	0.071	0.371 (0.206)
CFS	IMAT Index	51	0.153 (−0.075 to 0.380)	0.197	0.184	0.310 (0.233)
	SM Index	51	−0.032 (−0.089 to 0.025)	−0.179	0.269	0.301 (0.224)

Data are presented as unstandardised coefficients (B) with 95% confidence intervals, standardised coefficients (β), *p*-values and the effective sample size (n) for each model. Separate regression models were calculated for each outcome variable to compare the independent association of muscle quality (IMAT Index) with that of muscle quantity (SM Index). All models were adjusted for age, sex, BMI and ASA physical status as covariates and therefore contain five predictors. R^2^ values represent the proportion of variance explained by the full model including all covariates; adjusted R^2^ is given in parentheses. Variance inflation factors were below 2.2 in all models. Because of the small effective sample sizes, particularly for the TUG models (*n* = 25, corresponding to five observations per predictor), estimates are imprecise and should be interpreted as exploratory. *P*-values < 0.05 are considered statistically significant and are highlighted in bold.

#### 3.4.1 Prediction of muscle strength (handgrip)

The IMAT Index was independently associated with isometric handgrip strength. After adjustment for covariates, the IMAT Index remained significantly associated with handgrip performance (*B* = −1.808, 95% CI −2.994 to −0.622, *p* = 0.004, *n* = 42), indicating that a 1 cm^2^/m^2^ higher erector spinae IMAT Index corresponded to an approximately 1.8 kg lower handgrip strength. The full model explained 51.7% of the variance in handgrip strength (R^2^ = 0.517; adjusted R^2^ = 0.449). In contrast, when the quantitative SM Index was entered into the same adjustment model, it was not independently associated with handgrip strength (*B* = 0.062, 95% CI −0.268 to 0.393, *p* = 0.703, *n* = 42). Within the limits of this exploratory analysis, muscle quality therefore appears more relevant to strength than muscle quantity in this cohort.

#### 3.4.2 Prediction of functional mobility

For the TUG test, neither predictor was independently associated with performance. The SM Index showed an inverse association with TUG time that did not reach statistical significance (*B* = −0.293, 95% CI −0.614 to 0.028, *p* = 0.071, *n* = 25), and the IMAT Index was likewise not significantly associated with TUG performance (*B* = 0.993, 95% CI −0.336 to 2.322, *p* = 0.134, *n* = 25). These multivariable results are concordant with the univariate correlation analysis, in which neither index correlated with TUG performance (SM Index *ρ* = 0.133, *p* = 0.526; IMAT Index *ρ* = 0.115, *p* = 0.585). The TUG models were the most severely affected by missing data: functional testing was complete in only 26 of 53 patients, and after exclusion of one implausible segmentation result 25 observations remained for five predictors. The corresponding confidence intervals are wide, and no independent association between body composition and functional mobility can be established from these data.

#### 3.4.3 Prediction of frailty status

In the multivariable regression models for the CFS, neither the IMAT Index (*B* = 0.153, 95% CI −0.075 to 0.380, *p* = 0.184, *n* = 51) nor the SM Index (*B* = −0.032, 95% CI −0.089 to 0.025, *p* = 0.269, *n* = 51) was independently associated with frailty status in the total cohort. In these models the ASA score was the covariate most strongly associated with the CFS score, reflecting the global burden of comorbidity.

## 4 Discussion

### 4.1 Summary of principal findings

The present exploratory, retrospective, cross-sectional study investigated associations between automated CT-derived body composition parameters and clinical frailty assessments in a small cohort of geriatric patients undergoing preoperative evaluation. Our principal findings were threefold: First, in the total cohort the erector-spinae-based IMAT Index was associated with handgrip strength and remained so after adjustment for age, sex, BMI and ASA physical status, whereas its association with the CFS was borderline in univariate analysis and absent in multivariable analysis. The IMAT Index therefore behaved as a correlate of specific muscle weakness rather than of global clinical frailty (23, 24). Second, as hypothesised, CT-derived parameters showed their most pronounced associations with handgrip strength—a direct measure of muscle function—compared with more complex assessments such as the TUG or global judgements such as the CFS, reflecting that imaging metrics represent the morphological substrate of the muscle (12). Third, fully automated DLM-based segmentation proved feasible for deriving these parameters from routine CT imaging, indicating potential for the opportunistic identification of individuals at risk of physical decline without additional clinical burden (15, 22).

### 4.2 The role of myosteatosis in muscle weakness

The moderate independent association between the IMAT Index and handgrip strength observed in our cohort supports the hypothesis that myosteatosis is not merely a passive marker of aging, but an active contributor to muscle weakness in geriatric patients. The more pronounced association of IMAT with strength may reflect several pathophysiological mechanisms. First, IMAT acts as a physical disruptor of muscle architecture. Intramuscular fat infiltration directly impairs muscle contractile function by disrupting the spatial organization of muscle fibers and interfering with the lateral transmission of force required for efficient contraction (20). Furthermore, excessive IMAT accumulation can alter the pennation angle of contractile fibers, resulting in a mechanical disadvantage that compromises force production (17). Experimental evidence, such as in lipodystrophic mouse models, has further demonstrated that the presence of intramuscular fat directly impairs specific tension and contractile quality (19). Biochemically, IMAT functions as a metabolically active tissue capable of inducing local lipotoxicity and inflammation. Ectopic fat deposition within the muscle creates a pro-inflammatory microenvironment through adipokine secretion and immune cell infiltration, further compromising muscle function. Addison et al. reported that frail individuals exhibit significantly elevated levels of intramuscular IL-6, which correlate positively with IMAT volume (21). This suggests that IMAT serves as a paracrine signaling hub, secreting pro-inflammatory cytokines that may accelerate proteolysis and inhibit muscle anabolism in the immediate microenvironment. This inflammatory milieu may also contribute to systemic frailty through effects on other organ systems (24). Our findings are consistent with previous studies reporting that muscle quality metrics show closer associations with functional outcomes than muscle quantity. Jing et al. found that, after adjustment for confounders, muscle density and IMAT were independently associated with disability in activities of daily living, whereas the skeletal muscle index was not (24). Consequently, the measurement of IMAT may capture both the mechanical and metabolic deterioration of the muscle, which could explain its closer association with strength compared with muscle mass alone.

### 4.3 Dissociation between mass and function: the complexity of mobility

Our data did not reproduce a clear dissociation between muscle quality and muscle quantity across the different performance measures. While muscle quality (IMAT Index) was independently associated with isometric strength, neither muscle quality nor muscle quantity was independently associated with functional mobility as measured by the TUG test. Handgrip strength, as a direct measure of muscle force production, showed the most pronounced association with CT muscle parameters—particularly IMAT—representing a structure-function relationship in which imaging biomarkers relate to the physical property they anatomically represent. This finding regarding strength is consistent with the concept of dynapenia, described by Addison et al. and Cruz-Jentoft et al., in which the decline in muscle strength proceeds faster than the loss of muscle mass (21, 28). This dissociation suggests that qualitative factors, such as myosteatosis and neurological recruitment, contribute substantially to force generation. Our observation that neither index was independently associated with TUG performance is in line with previous reports: Borghi et al. found no correlation between muscle mass indices and dynamic balance tests, and Marcus et al. reviewed evidence that IMAT accumulation is associated with impaired mobility in older adults, without establishing muscle mass as the determining factor (31, 32). The absence of an association in our cohort likely reflects both the multifactorial nature of complex functional assessments and the very limited number of patients with valid TUG measurements (*n* = 25). In contrast to pure muscle force production, TUG performance and CFS scoring integrate multiple domains beyond skeletal muscle function—including balance, coordination, cognitive processing, cardiovascular fitness and, in the case of the CFS, comorbidity burden and functional independence. This does not diminish the clinical value of the TUG or the CFS but rather highlights that frailty is a syndrome not fully captured by any single biological parameter. Ultimately, our results suggest that fat infiltration of the erector spinae relates to pure muscle contraction (strength), but does not capture the global neurological, coordinative and comorbidity-related deficits that prolong TUG times or drive global clinical judgement.

### 4.4 Sexual dimorphism in body composition and frailty

In an exploratory sex-stratified analysis, correlations between adipose tissue indices (IMAT and VAT) and the CFS were observed in women but not in men. Because the female subgroup comprised only 20 participants, no sex-stratified regression was possible and no formal test for interaction was performed. The mechanistic considerations that follow are therefore offered as possible explanations for a hypothesis-generating observation rather than as evidence of a demonstrated sex-specific effect. One theoretical explanation for such a pattern would be the concept of physiological reserve together with hormonal differences. As proposed by Fried et al. and recently supported by Jiang et al., women possess a lower baseline of lean muscle mass compared to age-matched men (1, 33). Consequently, they operate closer to the frailty threshold. In this context of limited muscle reserve, the infiltration of ectopic fat (IMAT) likely exerts a relatively greater negative impact on the remaining functional muscle tissue. Even if absolute IMAT levels are comparable between sexes, the relative proportion of fat within the muscle compartment is higher in women, exacerbating functional decline (34). Furthermore, the distinct association of VAT with frailty in our female cohort reflects the metabolic consequences of estrogen withdrawal. As described by Soto et al. and Cornier et al., the menopausal transition triggers a redistribution of adipose tissue from subcutaneous to visceral and ectopic depots (35, 36). Hamrick et al. demonstrated that estrogen deficiency directly upregulates adipogenic gene expression in skeletal muscle (37). Our findings align with Laur et al., who identified that morphological changes like sarcopenia and myosteatosis are more strictly associated with frailty in women, whereas frailty in men may be driven by other mechanisms such as global tissue loss or weight loss (38). Whether postmenopausal women are indeed more susceptible to a “lipotoxic” phenotype of frailty cannot be determined from the present data and would require confirmation in adequately powered cohorts with formal tests of interaction.

### 4.5 Clinical utility: the era of AI-based opportunistic screening

Our findings have several important clinical implications. First, the association between IMAT and handgrip strength in the total cohort suggests that CT-derived muscle quality metrics could serve as objective biomarkers of specific physical vulnerability, such as reduced strength, rather than of global frailty, in geriatric patients who undergo CT imaging for other clinical indications (“opportunistic screening”) (23, 24). This approach requires no additional radiation exposure or patient burden beyond existing clinical care (14, 39). Second, the automated nature of the segmentation process makes this approach scalable and feasible for integration into routine clinical workflows (22, 40). Unlike manual segmentation, which often requires 20–30 min per scan and specialized expertise, automated DLM can process images in seconds with high precision and minimal human oversight (15, 22). This efficiency is crucial for translating research findings into clinical practice (41). Third, identification of high IMAT may represent a modifiable risk factor (11). While muscle mass increases slowly with resistance training (weeks to months), muscle quality may improve more rapidly through targeted interventions including exercise, nutritional optimization, and metabolic treatments aimed at reducing ectopic fat deposition (17, 34). This raises the possibility of personalized interventions guided by CT-derived metrics (28). Finally, these objective imaging biomarkers could serve as endpoints in clinical trials evaluating interventions to prevent or reverse frailty, offering more precise and reproducible measures than subjective clinical assessments alone (23, 41).

### 4.6 Limitations

This study has several limitations that warrant consideration. First, the study is exploratory and its cross-sectional, retrospective design precludes causal inference. We cannot determine whether IMAT accumulation contributes to frailty or whether frailty leads to increased IMAT through reduced physical activity and metabolic dysregulation. Longitudinal studies are needed to establish temporal relationships. Second, the sample size is small and selection bias cannot be excluded. Of 163 patients initially recruited at our centre, 64 were excluded because imaging or clinical data were unavailable, and a further 46 were excluded at eligibility assessment because the L3 vertebra or the full axial image was not within the scan range or because severe artifacts prevented reliable automated segmentation, leaving 53 patients (32.5% of those initially recruited). Patients excluded for incomplete L3 coverage or severe artifacts may differ systematically from those retained, for example with respect to body habitus, positioning ability or acute illness, which may limit generalisability. In addition, our sample was drawn from a single-centre surgical population undergoing perioperative evaluation and was predominantly of Caucasian European origin, further limiting applicability to community-dwelling older adults and to other ethnic groups and healthcare settings. Third, missing outcome data further reduced the effective sample size, most markedly for the TUG test, which was available in only 26 of 53 patients. As a consequence, the TUG regression models contained five predictors for 25 observations, which falls below conventional recommendations and carries a substantial risk of overfitting. The corresponding confidence intervals are wide and these estimates should be regarded as unstable. The sex-stratified analyses are similarly limited and were restricted to correlations. Fourth, while automated segmentation offers advantages in efficiency and reproducibility, its accuracy depends on the quality of the training data and may be affected by CT acquisition parameters, patient positioning and image artifacts. The reported validation metrics were obtained for the main tissue compartments in an unselected adult population and not specifically for IMAT delineation in geriatric patients. In the present cohort the automated muscle segmentation failed in one patient, producing a physiologically impossible value that was identified during visual quality control and set to missing. This illustrates that visual inspection of automated results remains necessary, and some degree of measurement error is inevitable. Fifth, we used single-slice CT analysis at L3, which is a validated approach but may not capture regional variations in muscle composition. Furthermore, while the SM Index encompassed all muscle groups at the L3 level, the IMAT Index was quantified exclusively within the erector spinae muscle. Although the erector spinae is an established representative trunk muscle for assessing myosteatosis, this measure is a surrogate and may not reflect intermuscular fat infiltration of the abdominal wall or psoas muscles. Sixth, our frailty assessments, while validated, have inherent limitations. Handgrip strength can be affected by acute factors (pain, motivation, arthritis), TUG performance depends on multiple systems beyond muscle, and the CFS involves subjective clinical judgement. Seventh, given the exploratory nature of the analysis and the number of associations examined, no correction for multiple testing was applied, so that individual *p*-values close to the conventional threshold should be interpreted with caution. Finally, we did not assess muscle radiodensity (mean Hounsfield units of muscle tissue), which has been proposed as another marker of muscle quality. Future studies should compare the relative value of IMAT, muscle radiodensity and other muscle quality metrics in adequately powered cohorts.

## 5 Conclusion

In this small, exploratory, retrospective and cross-sectional cohort of older surgical patients, an erector-spinae-based IMAT Index was associated with isometric handgrip strength and remained independently associated with it after adjustment for age, sex, BMI and ASA physical status. In contrast, the IMAT Index was not independently associated with global clinical frailty as measured by the CFS, and neither muscle quality nor muscle quantity was independently associated with functional mobility. Our findings therefore suggest that, in this cohort, CT-derived muscle quality relates to specific muscle weakness rather than to global frailty. Correlations between adipose tissue indices and the CFS were observed in women but not in men. Because the female subgroup was small, no sex-stratified regression or formal interaction test was performed, and these observations are hypothesis-generating only. Within these limitations, the results support the feasibility of automated, opportunistic CT analysis for extracting body composition biomarkers from routine imaging. Larger, adequately powered and preferably longitudinal studies are needed to confirm these associations, to test for sex-specific effects and to determine whether reducing IMAT through targeted prehabilitation influences functional outcomes.

## Data Availability

The original contributions presented in the study are included in the article, further inquiries can be directed to the corresponding author/s.
